# Factors associated with increased costs in robotic gastric bypass surgery: Australian healthcare system perspective

**DOI:** 10.1007/s11701-025-02483-2

**Published:** 2025-07-03

**Authors:** Marianne Huynh, I.-Wen Pan, Matthew Kroh

**Affiliations:** 1 Medtronic, Macquarie Park, Australia; 2 Medtronic, Boston, MA USA; 3 Cleverland Clinic Lerner College of Medicine, Cleveland, OH USA

**Keywords:** Robotic surgery, Roux-en-Y gastric bypass, Inpatient cost, Robotic stapler, Laparoscopic stapler

## Abstract

This study aimed to understand the cost drivers of robotic Roux-en-Y gastric bypass (rRYGB) with specific application to the Australian healthcare system. US-based PINC AI™ Healthcare Data (1/1/2021–12/31/2022) were used to extract patients who underwent elective rRYGB procedures. We included patients who used single-type staplers in each case, had non-zero costs, and non-missing key variables. Factors including patient and hospital characteristics, type of staplers used [laparoscopic bedside staplers (LBS), other unspecified bedside staplers (OBS), and robotic staplers (RS)] were evaluated. Multivariable general linear models were used to identify cost drivers. Sensitivity analysis was done by the bootstrapping method. The results were then applied to Australian healthcare system model. In this US cohort, 7606 rRYGB discharges were studied, including 18.9% LBS, 8.7% OBS, and 72.4% RS cases. The factors associated with increased costs included type of staplers, patients older than 54, male, non-Hispanic White, diagnosis of obesity, comorbidity >  = 3, severity of illness, hospitals in the Northeast, located in rural area, with large size (> = 500 beds), lower surgeon annual volume, and procedures done in 2022. After adjusting for other factors, compared to OBS and RS, rRYGB used the LBS significantly reduced total inpatient cost by $2,220 ± $432 (Mean difference ± Standard deviation) and $2,119 ± $181, and significantly reduced operative-room time by 43.3 ± 3.9 and 41.4 ± 2.1 min, respectively. Meanwhile, LBS has equivalent outcomes, including blood transfusion, bleeding, anastomotic leak, and ICU visits, compared to other types of staplers. RS and OBS were essential factors associated with increased costs in patients treated with rRYGB compared to LBS.

**Supplementary Information:**

The online version contains supplementary material available at 10.1007/s11701-025-02483-2.

## Introduction

Published literature, including systematic reviews, has shown robotic bariatric procedures have similar or controversial clinical outcomes, with potentially higher costs compared to the laparoscopic approach [1–5]. However, the adoption of robotic Roux-en-Y gastric bypass (rRYGB) continues to rise steadily [6, 7].

Similarly, since introducing surgical robotic systems into the Australian healthcare system in 2003, there has been an exponential uptake of robotic procedures by various surgical specialties. Nevertheless, several factors that impeded robotic surgery uptake in the Australia public sector include costs, training, and availability. A study reported that total hospital costs of robot-assisted procedures were significantly higher than other surgical modalities and there are no cost offsets if provided in Australian public hospitals, though the robotic procedures have demonstrated a shorter inpatient length of stay and quicker speed of recovery [8]. This is attributed to the costs of robotic-assisted procedures are more than the activity-based funding (ABF) revenue, and the robot platform is an additional capital cost.

Moreover, the evidence for robot-assisted bariatric surgery as a superior surgical option is inconclusive, and evidence around the cost-effectiveness of robot-assisted surgery is limited within the Australian context. More high-quality, long-term studies are needed to confirm the comparative clinical effectiveness of robotic surgery over conventional/laparoscopic surgical procedures.

Bedside stapling was required for robotic-assisted bariatric operations prior to the introduction of the first generation of Intuitive endoscopic staplers in 2014. Clinical case studies have shown that robotic staplers require more recharges and operating room time and are more costly than bedside stapling during robotic gastric bypass or other bariatric procedures [9, 10].

There is a lack of real-world evidence generated in investigating the factors associated with increased costs of rRYGB. The objective of this study is to understand the cost drivers of rRYGB and focus on the cost variations associated with different types of staplers by using the US hospital-level database and subsequent application to the rapidly evolving robotic surgery practices in Australia.

## Methods

### Data sources

The PINC AI™ Healthcare Data (PHD) was used in the study. PHD includes “hospital-based, service-level information on inpatient discharge”; outpatient visits to emergency departments, ambulatory surgery centers, and alternate sites of care are also included. It represents “approximately 25% of annual United States inpatient admissions” [11]. Hospital and visit information, physician specialties, healthcare payers, and patient data, including demographics, disease states, diagnoses, costs, medications, and device details from standard charge master and hospital original charge files, are included in the PHD. Patient and provider information in the PHD is de-identified and HIPAA-compliant in accordance with the HIPAA Privacy Rule. The Sterling Institutional review board (IRB) determined that this study was qualified an exemption from full board review.

### Study cohort

**Fig. 1:**
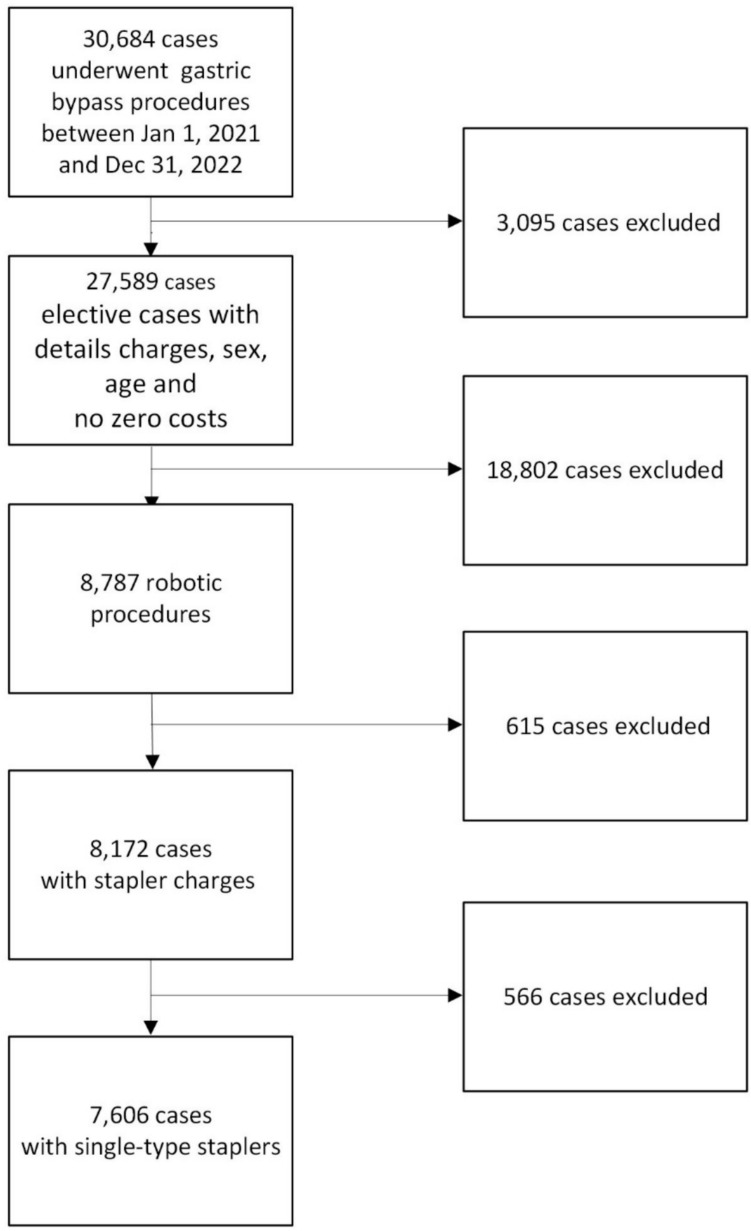
Flow chart of study cohort selection

Patients admitted to hospital inpatient setting and underwent elective primary gastric bypass procedures with a robotic platform between 1/1/2021 and 12/31/2022, used any single-type surgical stapler, had non-missing sex and age variables, and had non-zero costs (Fig. 1). The robotic procedures were determined if met any following requirements, 1) secondary inpatient procedure claim with a ICD-10 procedure code ‘8E0W4CZ’ indicated robotic surgery, 2) any claim with a Current Procedure Terminology code ‘S2900’ indicated robotic surgery, 3) patients charged with robotic supplies (searching text “ROBOTIC” through charge files).

### Study design

#### Patients’ characteristics and type of staplers used during rRYGB procedures

Three patients’ characteristics were divided into four groups: age: under 40, 40–54, 55–64, or 65 and older; race and ethnicity: Non-Hispanic White or Black, Hispanic, or others; insurer or payer: Medicare, Medicaid, private/commercial insurer, or other payers (including self-pay). The other dichotomous patient variables included sex (male, female), patients with a primary obesity diagnosis (ICD-10 diagnosis code: E66.01, morbid (severe) obesity due to excess calories) or no indicated obesity diagnosis, concurrent Charlson comorbidity index (CCI) score (0–2, or 3 and above) [12], and severity of illness [all patients refined diagnosis-related group (APR-DRG) severity of illness subclasses: minor, or others (moderate/major/extreme)].

We categorized surgical staplers into three groups: robotic staplers (RS) included Intuitive Sureform™ and Endowrist™ staplers; laparoscopic bedside staplers (LBS) included Medtronic Signia™, Endo-GIA™, Tri-staple™, and Johnson and Johnson Ethicon®, and Echelon™ staplers; and other bedside staplers (OBS) included Lexington AEON™ and other unspecified non-robotic staplers. The stapler was identified by using product names or numbers, or a combination of product names and numbers, through text searching of the hospital charge master file.

#### Provider characteristics

The provider characteristics included hospital census region of Northeast, Midwest, South, or West, hospital located in urban or rural area, hospital bed size under 300 beds, 300–499 beds, or equal or larger than 500 beds, and teaching status. The provider volume included hospitals’ and surgeons’ volume, which were dichotomized by hospitals’/surgeons’ bariatric procedure volume annually, greater than the 75th percentile of the volume defined as high volume, less or equal to the 75th percentile of the volume defined as low volume.

#### Hospitalized costs

Three hospitalized cost variables were derived from PHD: total variable cost, total fixed cost, and total inpatient cost (the sum of total variable and fixed cost). The total variable cost consisted of the expenses directly related to or varying with the department’s activity (volume), such as medical supplies, medications, and hands-on patient care. Other expenses treated the patient during the hospitalization but not related directly or were various with the department’s activity, such as depreciation, repair costs, maintenance costs, overhead, and management/administrative related costs were included in the total fixed costs. To adjust for inflation, 2021 cost data were adjusted to 2022 US dollars by the consumer price index [13].

#### Operating room (OR) time and clinical outcomes

OR time in minutes was obtained from the hospital charge file in PHD. The clinical outcomes included the incidence rates of anastomotic leak, bleeding, blood transfusion, and intensive care unit (ICU) visits, which were identified perioperatively or postoperatively during the hospitalization.

The Supplemental Table included all study codes for identifying procedures, diagnoses, and clinical outcomes.

### Statistical analysis

Given a required significance level of 0.05 and statistical power 80%, the smallest sample size needed for each comparison group (LBS, OBS, and RS) for the study is 347. Factors including patients and hospital characteristics, and type of staplers used were evaluated. Descriptive analysis reported the number of cases and proportion by subcategory for categorical variables which include patient demographics and clinical characteristics. The mean, standard deviation of the unadjusted costs, and OR time were reported.

Bivariate analysis such as chi-square or Fisher exact test, and t-test or ANOVA were used to examine the baseline balance and cost variations among covariates subcategories.

Multivariable general linear models identified cost drivers of gastric bypass surgical procedures. Gamma or binomial distribution and log-link function were used in the general linear models with respective costs/operating room time or clinical outcomes. The adjusted costs and other outcomes were reported. Bootstrapping method was used for sensitivity analysis to test the robustness of the multivariable general linear models. The statistical significance was determined if p-value <  = 0.05. All data management and analyses were done by using SAS 9.4 (SAS Institute Inc., Cary, NC) and Stata 18.0 (StataCorp, College Station, TX) using 2-sided statistical tests.

## Results

A total of 7606 discharges (Fig. 1) were eligible for study inclusion. Among them, 1436 (18.9%) LBS, 659 (8.7%) OBS, and 5511 (72.4%) RS cases. The majority of patients were age 40 to 54 (44.4%), female (85.5%), non-Hispanic white (55.3%), with private insurer (55.4%), obesity diagnosis (88.6%), CCI < 3 (92.1%), minor disease severity (78.2%), treated at hospitals in South region (42.9%), urban (95.6%), with large bed size (500 beds and up) (41.4%), teaching (62.4%), having high bariatric procedure volume (60.2%), and the procedures done by surgeons with high bariatric procedure volume (71.1%). Significant variations exist among the three types of staplers in patient and hospital characteristics, except for comorbidities and surgeons’ bariatric procedure volume. (Table 1).

**Table 1:** Baseline patient and provider information

	All patients		LBS (*N* = 1436)	OBS (*N* = 659)	RS (*N* = 5511)	*p*-value
Description	N	%	%	%	%	
Total	7606		18.88	8.66	72.46	
Age (year)						0.001^§^
Under 40	2570	33.79	32.94	34.29	33.95	
Between 40 and 54	3380	44.44	45.89	39.00	44.71	
Between 55 and 64	1237	16.26	16.09	17.60	16.15	
Older than 65	419	5.51	5.08	9.10	5.19	
Sex						0.007^§^
Female	6503	85.50	86.77	81.64	85.63	
Male	1103	14.50	13.23	18.36	14.37	
Race and ethnicity						< 0.001^§^
White (non-Hispanic)	4207	55.31	62.26	54.63	53.58	
Black (non-Hispanic)	1353	17.79	20.61	12.44	17.69	
Hispanic	1272	16.72	7.45	22.00	18.51	
Others	774	10.18	9.68	10.93	10.22	
Insurer or payer						< 0.001^§^
Medicare	1069	14.05	13.72	13.51	14.21	
Medicaid	1805	23.73	17.41	22.61	25.51	
Private or commercial insurer	4210	55.35	61.56	56.60	53.58	
Other	522	6.86	7.31	7.28	6.70	
Primary obesity						< 0.001^§^
Yes	6741	88.63	91.78	85.89	88.13	
No	865	11.37	8.22	14.11	11.87	
Charlson comorbidity index scores						0.854
0–2	7001	92.05	91.85	92.56	92.03	
3 +	605	7.95	8.15	7.44	7.97	
Severity of illness						0.070
Minor	5950	78.23	80.01	79.97	77.55	
Others	1656	21.77	19.99	20.03	22.45	
Hospital census region						< 0.001^§^
Northeast	1769	23.26	18.38	41.73	22.32	
Midwest	1593	20.94	28.27	0.76	21.45	
South	3259	42.85	47.91	46.13	41.14	
West	985	12.95	5.43	11.38	15.10	
Hospital location						< 0.001^§^
Rural	333	4.38	0.70	16.08	3.94	
Urban	7273	95.62	99.30	83.92	96.06	
Hospital bed size						< 0.001^§^
Under 300 beds	2688	35.34	34.05	5.46	39.25	
Between 300 and 499	1772	23.30	14.42	26.10	25.28	
500 and above beds	3146	41.36	51.53	68.44	35.47	
Hospital teaching status						< 0.001^§^
No teaching	2857	37.56	51.81	5.01	37.74	
Teaching	4749	62.44	48.19	94.99	62.26	
Hospital bariatric procedure volume						< 0.001^§^
Low (< = 75th percentile)	3029	39.82	28.90	40.52	42.59	
High (> 75th percentile)	4577	60.18	71.10	59.48	57.41	
Surgeon bariatric procedure volume						0.403
Low (< = 75th percentile)	2195	28.86	27.86	27.47	29.29	
High (> 75th percentile)	5411	71.14	72.14	72.53	70.71	
Year of the procedure						< 0.001^§^
2021	3547	46.63	53.55	31.11	46.69	
2022	4059	53.37	46.45	68.89	53.31	

*LBS* laparoscopic bedside staplers; *OBS* other bedside staplers; *RS* robotic staplers; ‘§’ showed statistically significant

The average total inpatient cost, variable cost, and fixed cost per robotic gastric bypass were $18,117 (standard deviation: $10,506), $10,133 ($5627), and $7984 ($6159), respectively. The cost drivers/factors associated with increased costs of total inpatient costs included type of staplers, patients >  = 55 years of age, male, with non-Hispanic Black, or Hispanic, with non-primary obesity diagnosis, higher Charlson comorbidity index scores (> = 3), higher disease severity (moderate to Extreme severity), and treated at the hospitals in Northeast region, rural, 500 + beds, no teaching, and/or treated by surgeons with lower volume, and/or procedures were done in 2022. (Table 2, all *p* values < 0.05). Factors associated with rising total variable costs were similar to total inpatient costs, except hospitals in the West region and/or those with higher volume were likely to have higher total variable costs. For total fixed costs, type of staples was also a key cost driver. Also, patients >  = 65 years of age, with non-Hispanic Black, or Hispanic, with non-obesity diagnosis, CCI >  = 3, higher disease severity, treated at hospitals in Northeast, rural, with large size (500 beds and more), with teaching status, and lower hospital and surgeon volume would likely have higher total fixed costs.

**Table 2:** Cost estimation using multivariable generalized linear model

	Total inpatient costs				Total variable costs				Total fixed costs			
Covariates	Coefficient	95% CI		*p*-value	Coefficient	95% CI		*p*-value	Coefficient	95% CI		*p*-value
Type of staplers												
LBS	Reference				Reference				Reference			
OBS	0.13	0.08	0.17	***^§^	0.13	0.08	0.18	***^§^	0.18	0.12	0.23	***^§^
RS	0.12	0.10	0.14	***^§^	− 0.02	− 0.04	0.00	0.02^§^	0.40	0.36	0.45	***^§^
AGE												
Under 40	Reference				Reference				Reference			
Between 40 and 54	0.02	0.00	0.04	0.13	0.01	− 0.02	0.03	0.57	0.03	0.00	0.06	0.07
Between 55 and 64	0.03	0.00	0.06	0.03^§^	0.04	0.01	0.07	0.01^§^	0.02	− 0.02	0.06	0.34
Older than 65	0.11	0.05	0.17	***^§^	0.11	0.05	0.17	***^§^	0.10	0.02	0.18	0.01^§^
Sex												
Female	Reference				Reference							
Male	0.04	0.00	0.07	0.03^§^	0.04	0.01	0.07	0.01^§^	NA	NA	NA	NA
Race and ethnicity												
White (non-Hispanic)	Reference				Reference				Reference			
Black (non-Hispanic)	0.05	0.02	0.07	***^§^	0.03	0.00	0.05	0.02^§^	0.05	0.01	0.08	0.02^§^
Hispanic	0.07	0.04	0.09	***^§^	0.06	0.03	0.09	***^§^	0.07	0.03	0.12	***^§^
Others/Unknown	0.08	0.04	0.11	***^§^	0.09	0.05	0.13	***^§^	0.06	0.02	0.11	0.01^§^
Primary obesity												
Yes	Reference				Reference				Reference			
No	0.12	0.09	0.16	***^§^	0.10	0.07	0.14	***^§^	0.16	0.11	0.21	***^§^
Charlson Comorbidity Index scores												
0–2	Reference				Reference				Reference			
3 +	0.09	0.04	0.14	***^§^	0.08	0.03	0.13	***^§^	0.11	0.04	0.17	***^§^
Severity of illness												
Minor	Reference				Reference				Reference			
Others	0.18	0.15	0.20	***^§^	0.16	0.13	0.19	***^§^	0.21	0.17	0.25	***^§^
Hospital Census Region												
Northeast	Reference				Reference				Reference			
Midwest	− 0.10	− 0.13	− 0.08	***^§^	− 0.03	− 0.06	0.00	0.05^§^	− 0.18	− 0.22	− 0.14	***^§^
South	− 0.17	− 0.20	− 0.14	***^§^	− 0.10	− 0.13	− 0.07	***^§^	− 0.33	− 0.37	− 0.29	***^§^
West	− 0.05	− 0.09	− 0.02	0.01^§^	0.09	0.05	0.13	***^§^	− 0.22	− 0.28	− 0.17	***^§^
Hospital location												
Rural	Reference				Reference				Reference			
Urban	− 0.37	− 0.44	− 0.30	***^§^	− 0.30	− 0.37	− 0.23	***^§^	− 0.51	− 0.58	− 0.43	***^§^
Hospital Bed Size												
Under 300 beds	Reference				Reference				Reference			
Between 300 and 499	− 0.05	− 0.08	− 0.02	***^§^	− 0.09	− 0.12	− 0.07	***^§^	0.02	− 0.02	0.06	0.35
500 and above beds	0.13	0.10	0.16	***^§^	0.08	0.05	0.10	***^§^	0.25	0.21	0.30	***^§^
Hospital teaching status												
No teaching	Reference				Reference				Reference			
Teaching	− 0.11	− 0.13	− 0.08	***^§^	− 0.23	− 0.25	− 0.21	***^§^	0.08	0.05	0.12	***^§^
Hospitals’ volume												
Low (< = 75th percentile)					Reference				Reference			
High (> 75th percentile)	NA	NA	NA	NA	0.07	0.05	0.09	***^§^	− 0.16	− 0.19	− 0.13	***^§^
Surgeons’ volume												
Low (< = 75th percentile)	Reference				Reference				Reference			
High (> 75th percentile)	− 0.08	− 0.11	− 0.06	***^§^	− 0.05	− 0.08	− 0.03	***^§^	− 0.10	− 0.14	− 0.06	***^§^
Procedure Year												
2021	Reference				Reference							
2022	0.03	0.01	0.05	0.01^§^	0.04	0.02	0.06	***^§^	NA	NA	NA	NA

All models were parsimony models; ***: p−value <0.001; ‘§’ showed statistically significant; *NA* not applicable; *CI* confidence interval; *LBS* laparoscopic bedside staplers; *OBS* other bedside staplers; *RS* robotic staplers

After adjusting other factors associated with increased costs, the LBS significantly reduced total inpatient cost by $2220 (95% confidence interval (CI): $1373 ~ $3067) and $2119 (95% CI: $1765 ~ $2473) compared to OBS and RS, respectively. Meanwhile, RS cases had the lowest variable (estimated mean ± standard error: $9942 ± $62 USD) and highest fixed costs ($8742 ± $79 USD) among the three types of staplers (Table 3).

**Table 3:** Cost estimation: unadjusted, adjusted, and the difference between type of staplers

Type of costs	Total inpatient costs	*P*-value	Variable costs	*P*-value	Fixed costs	*P*-value
Unadjusted costs	$18,117($10,506)		$10,133($5627)		$7984($6159)	
Type of staplers	Mean (STD)		Mean (STD)		Mean (STD)	
LBS	$16,107 ($5,891)	< 0.001^§^	$10,191 ($2918)	< 0.001^§^	$5,916 ($4466)	< 0.001^§^
OBS	$20,615 ($24,373)		$11,634 ($12,101)		$8981 ($12,791)	
RS	$18,342 ($8398)		$9938 ($4867)		$8404 ($5119)	
Adjusted costs						
Type of staplers	Mean (SE)		Mean (SE)		Mean (SE)	
LBS	$16,369 ($157)	< 0.001^§^	$10,164 ($81)	< 0.001^§^	$5838($110)	< 0.001^§^
OBS	$18,589 ($416)		$11,589 ($301)		$6965($151)	
RS	$18,488 ($110)		$9942 ($62)		$8742($79)	
Differences between type of staplers	Mean (95%CI)		Mean (95%CI)		Mean (95%CI)	
OBS minus LBS	$2220 ($1373, $3067)	< 0.001^§^	$1425 ($823, $2027)	< 0.001^§^	$1127 ($780, $1473)	< 0.001^§^
RS minus LBS	$2,119 ($1765, $2473)	< 0.001^§^	$-222 ($-416, $-28)	0.02^§^	$2904 ($2626, $3181)	< 0.001^§^
RS minus OBS	$-101 ($-910, $708)	0.806	$-1,647 ($-2240, $-1054)	< 0.001^§^	$1777 ($1462, $2092)	< 0.001^§^

*STD* standard deviation; *SE* standard error; *CI* confidence interval; *LBS* laparoscopic bedside stapler; *OBS* other bedside stapler; *RS* robotic stapler; ‘§’showed statistically significant

In addition to costs, the OR time in LBS group was the shortest; LBS reduced 43.3 ± 3.9 and 41.4 ± 2.1 min compared to OBS and RS. (Table 4) We also observed that LBS has equivalent outcome performance, including blood transfusion, bleeding, anastomotic leak, and ICU visits, compared to OBS and RS (Table 4). Sensitivity analysis showed similar results.

**Table 4:** Adjusted operating room time and incidence rates of blood transfusion, bleeding, anastomotic leak, and intensive care unit (ICU) visit

Type of stapler	Adjusted outcomes (95% confidence interval)			RS vs LBS (Difference = RS minus LBS)		OBS vs LBS (Difference = OBS minus LBS)	
Outcomes	LBS	OBS	RS	Mean difference (95% CI)	*p*-value	Mean difference (95% CI)	*p*-value
Operating room time (minutes)	164.7 (162, 167.5)	208 (201.6, 214.5)	206.1 (203.6, 208.7)	41.4 (37.6, 45.1)	< 0.001^§^	43.3 (36.1, 50.5)	< 0.001^§^
Bleeding (%)	2.2 (1.5, 3.0)	2.8 (1.3, 4.2)	1.9 (1.6–2.3)	− 0.33 (− 1.18, 0.52)	0.427	0.53 (− 1.17, 2.23)	0.527
Blood transfusion (%)	0.5 (0.1, 0.9)	0.6 (0.0, 1.2)	0.7 (0.5–0.9)	0.16 (− 0.30, 0.61)	0.534	0.09 (− 0.65, 0.82)	0.816
Anastomotic leak (%)	0.3 (0.02, 0.65)	0.8 (0.13, 1.46)	0.4 (0.22, 0.54)	0.05 (− 0.30, 0.40)	0.793	0.46 (− 0.25, 1.17)	0.162
ICU visit (%)	0.4 (0.02, 0.75)	1.1 (0.28, 1.89)	0.7 (0.51, 0.92)	0.33 (− 0.10, 0.75)	0.221	0.70 (− 0.16, 1.56)	0.081

*LBS* laparoscopic bedside stapler; *RS* robotic staplers; *OBS* other unspecified staplers; *CI* confidence interval;’§’ showed statistically significant

## Discussion

This important study demonstrated that RS and OBS staplers used in robotic gastric bypass procedures are key cost drivers, along with other patient and hospital characteristics, in the US hospital setting. Compared to RB and OBS, LBS would save approximately $2200 USD and reduce 41 min in operating room time per rRYGB while providing equivalent safety features, including similar incidence rates of blood transfusion, bleeding, anastomotic leak, and ICU visits. The surgical practices in bariatric procedures are similar across different healthcare systems and countries [14]. Therefore, these study results may be reflected in the Australian healthcare system and have important specific implications for economic efficiency of care delivery. 

According to the Royal Australasian College of Surgeons report, as of May 2023, 136 (84%) of the 162 robotic platforms in Australia are in private hospitals, which limits access for most Australians without private insurance [15]. This is because health services are provided with the same funding amount based on the nationally efficient price for the procedure code in public hospitals, which provides little or no incentive for robotic surgery [16, 17]. However, the financial impacts of the widespread use of robotic surgery in Australia are significant, with approximately $AUD4.5 million for capital, engineering work, and compatible equipment upgrades. Ongoing costs include consumables of up to $AUD5000 per patient and annual servicing costs of $AUD250,000 [16]. Various clinical pathways, including bedside stapling, can be explored to ensure sustainable funding for robot-assisted bariatric surgery in Australia and worldwide. In this study, we found that the type of staplers used in rRYGB, as well as a variety of patient and hospital characteristics, were associated with the increased cost of robotic gastric bypass procedures. Specifically, laparoscopic bedside staplers were cost-saving and efficient in robotic gastric bypass procedures compared to robotic staplers and other non-specified bedside staplers.

A previous study observed a similar pattern of cost and OR time when using staplers for robotic gastric sleeve procedures: the LBS saved $2,700 USD and 11 min OR time per case compared to RS [18]. Unlike gastric sleeve procedures, which largely rely on mechanical staplers, gastric bypass procedures are often done with surgical staplers and other medical devices, such as radiofrequency or ultrasonic energy devices. As a result, the larger scale of time savings of LBS compared to RS may not only be contributed by staplers but also to other factors that need further investigation. Two studies investigating the costs and outcomes of LBS and RS used in robotic gastric bypass based on institutional clinical data provided insight into the cost and OR time variations [9, 19]. Both studies showed that LBS reduced OR time (reduced 18 and 22 min), number of stapler recharges, and total inpatient costs of stapler per case ($425 USD and $697 USD) and no difference in clinical outcomes compared to RS. One study also indicated that fewer instrument changes may contribute to reducing OR time. Practically, using bedside staplers in robotic gastric bypass requires two surgeons or one surgeon and a well-trained assistant to perform the procedures. In some cases, for example, some states in the US allow trained physician assistants or nurse practitioners to perform the stapling under a surgeon’s supervision. In Australia, it is unknown if bedside stapling would require additional costs due to the changes in practice patterns.

A study found that 37% of the bariatric costs were attributable to hospital-level factors [20]. In this study, several providers’ factors were also identified as associated with increased total hospital costs: Northeast region, large hospital (500 + beds), rural, non-teaching, and low volume surgeons. The patient-level factors such as being male and having multiple comorbidities have also been identified in a prior study that are associated with higher bariatric costs [21]. There are slight differences in factors associated with total inpatient costs, variable costs, and fixed costs. However, limited information regarding the details of each cost type prevents further exploration of the rationale for cost decomposition. There are limitations to this study due to the essence of the claim database, compared to the data collected from clinical practice. The hospital-based claim database does not include the granularity of staple load (height) or the presence of staple line reinforcement and the details of the costs; for example, it is unmeasurable for the number or the size of stapler reloads used in each procedure. Therefore, we could not accurately identify if there are cost differences in stapler supplies or the other variables, as well as the fixed costs. Also, the Da Vinci robotic system remains dominant in the US robotic surgery market, while Australia has been introducing other robotic surgical systems, which may increase the complexity of cost variation compared to the US healthcare system. In addition, the healthcare funding mechanisms for surgical procedures differ in the Australian public and private hospital systems, as previously mentioned and the US healthcare system. In the US, inpatient surgical procedures are based on the DRG system and other factors or fees for services as well as funding resources/payers. Data included here are modeled after the US healthcare system and applied to the Australian healthcare system, and therefore, may not include all variables of either system and subsequent accuracy of analysis. Also noted, the authors’ conflict of interest, which may introduce a potential bias, has been disclosed along with this manuscript.

## Conclusion

The staplers integrated into the robotic platform and other named or unnamed non-robotic staplers are essential factors associated with increased costs in patients treated with rRYGB compared to the major-branded bedside staplers. Without clinical outcome compromise and effectiveness, choosing staplers used in gastric bypass procedures performed in robotic platform could help reduce resource consumption when resources are limited and allow more widespread adoption of robotic surgical technologies at a lower cost. Future studies exploring the concept of robot-assisted bariatric surgery with bedside stapling in the Australian healthcare system to ensure longer-term clinical benefits and attendant cost savings are warranted.

## Supplementary Information

Below is the link to the electronic supplementary material.

Supplementary file1 (PDF 63 KB)

## Data Availability

This study used third party data—PINC AI™ Healthcare Data- made available under a license that the authors does not have permission to share. Requests to access the data should be directed to Premier at https://premierinc.com/
