# Supplementary material for: Factors associated with increased costs in robotic gastric bypass surgery: Australian healthcare system perspective
Source: J Robot Surg. 2025 Jul 3;19(1):344. doi: 10.1007/s11701-025-02483-2 (PMC12226609; doi:10.1007/s11701-025-02483-2)
Supplement: Supplementary file 1 — Supplementary file1 (PDF 63 KB) [file 11701_2025_2483_MOESM1_ESM.pdf]

Title: Factors associated with rising costs in robotic gastric bypass surgery – Australian healthcare system perspective

Journal: Journal of Robotic Surgery

Marianne Huynh[1], I-Wen Pan[2], Matthew Kroh[2]

[1]Medtronic, Macquarie Park, Australia, [2]Medtronic, Boston, USA

Corresponding author: Matthew Kroh, Email: krohm@ccf.org

Supplemental Table:

Table: study code list

Variable defined	Codes	Type of codes
gastric bypass	0D164ZA	ICD 10 PCS
obesity	E66.01	ICD 10 DX
blood transfusion	30233H0, 30233N0, 30243H0, 30243N0, 30233H1, 30243H1, 30233N1, 30233P1, 30243N1, 30243P1, 30230H0, 30230H1, 30230J0, 30230J1, 30230K0, 30230K1, 30230L0, 30230L1, 30230M0, 30230M1, 30230N0, 30230N1, 30230P0, 30230P1, 30230Q0, 30230Q1, 30230R0, 30230R1, 30230S0, 30230T0, 30230V0, 30230W0, 30233J0, 30233J1, 30233K0, 30233K1, 30233L0, 30233L1, 30233M0, 30233M1, 30233P0, 30233Q0, 30233Q1, 30233R0, 30233R1, 30233S0, 30233T0, 30233V0, 30233W0, 30240H0, 30240H1, 30240J0, 30240J1, 30240K0, 30240K1, 30240L0, 30240L1, 30240M0, 30240M1, 30240N0, 30240N1, 30240P0, 30240P1, 30240Q0, 30240Q1, 30240R0, 30240R1, 30240S0, 30240T0, 30240V0, 30240W0, 30243J0, 30243J1, 30243K0, 30243K1, 30243L0, 30243L1, 30243M0, 30243M1, 30243P0, 30243Q0, 30243Q1, 30243R0, 30243R1, 30243S0, 30243T0, 30243V0, 30243W0, 30250H0, 30250H1, 30250J0, 30250J1, 30250K0, 30250K1, 30250L0, 30250L1, 30250M0, 30250M1, 30250N0, 30250N1, 30250P0, 30250P1, 30250Q0, 30250Q1, 30250R0, 30250R1, 30250S0, 30250T0, 30250V0, 30250W0, 30253H0, 30253H1, 30253J0, 30253J1, 30253K0, 30253K1, 30253L0, 30253L1, 30253M0, 30253M1, 30253N0, 30253N1, 30253P0, 30253P1, 30253Q0, 30253Q1, 30253R0, 30253R1, 30253S0, 30253T0, 30253V0, 30253W0, 30260H0, 30260H1, 30260J0, 30260J1, 30260K0, 30260K1, 30260L0, 30260L1, 30260M0, 30260M1, 30260N0, 30260N1, 30260P0, 30260P1, 30260Q0, 30260Q1, 30260R0, 30260R1, 30260S0, 30260T0, 30260V0, 30260W0, 30263H0, 30263H1, 30263J0, 30263J1, 30263K0, 30263K1, 30263L0, 30263L1, 30263M0, 30263M1, 30263N0, 30263N1, 30263P0, 30263P1, 30263Q0, 30263Q1, 30263R0, 30263R1, 30263S0, 30263T0, 30263V0, 30263W0, 3E030GC, 3E033GC, 3E040GC, 3E043GC, 3E050GC, 3E053GC, 3E060GC, 3E063GC,	ICD 10 PCS
	36430, 86890, 86891, 86985, P9010, P9011, P9012, P9016, P9017, P9019, P9020, P9021, P9022, P9023, P9032, P9033, P9034, P9035, P9036, P9037, P9038, P9039, P9040, P9043, P9044, P9048, P9050, P9051, P9052, P9053, P9054, P9055, P9056, P9057, P9058, P9059, P9060, P9070, P9071, P9072,	CPT
bleeding	D62, R58, K92.2, K92.1, K91.840, K91.841, K91.870, K91.871, N99.62, N99.820, N99.821,	ICD 10 DX
anastomotic leak	K63.2, K65.1, K91.3, K91.89, K91.81, K91.83	ICD 10 DX
